# Tumoral and normal brain tissue extraction protocol for wide-scope screening of organic pollutants

**DOI:** 10.1016/j.mex.2023.102069

**Published:** 2023-02-10

**Authors:** Daniel Gutiérrez-Martín, Montse Marquès, Albert Pons-Escoda, Noemi Vidal, Jordi Bruna, Esteban Restrepo-Montes, Rebeca López-Serna, Francisco García-Sayago, Carles Majos, Pablo Gago-Ferrero, Rubén Gil-Solsona

**Affiliations:** a Institute of Environmental Assessment and Water Research (IDAEA), Severo Ochoa Excellence Center, Spanish Council for Scientific Research (CSIC), Jordi Girona 18-26, E-08034 Barcelona, Spain; b Universitat Rovira i Virgili, Laboratory of Toxicology and Environmental Health, School of Medicine, Sant Llorenç 21, 43201 Reus, Catalonia, Spain; c Institut d'Investigació Sanitària Pere Virgili (IISPV), Reus, Spain; d Institut de Diagnóstic per la Imatge (IDI), Department of Radiology, Hospital de Bellvitge, 08907 L'Hospitalet de Llobregat, Barcelona, Spain; e Neuro-Oncology Unit, Hospital Universitari de Bellvitge-ICO L'Hospitalet, and Institut d'Investigació Biomèdica de Bellvitge (IDIBELL), 08907 L'Hospitalet de Llobregat, Barcelona, Spain; f Deparment of Pathology, Hospital de Bellvitge, 08907 L'Hospitalet de Llobregat, Barcelona, Spain; g Institute of Neurosciences, Department of Cell Biology, Physiology, and Immunology, Autonomous University of Barcelona, Biomedical Research Networking Center on Neurodegenerative Diseases (CIBERNED), Bellaterra, Spain; h Department of Analytical Chemistry, Faculty of Sciences, University of Valladolid, 47011 Valladolid, Spain; i Institute of Sustainable Processes, Dr. Mergelina s/n, Valladolid 47011, Spain; j Institut de Medicina Legal de Catalunya i Ciències Forenses, Departament de Justícia, Tarragona, Spain

**Keywords:** Human tissue, Non-target screening, Suspect screening, Bead beating, LC-HRMS, Human biomonitoring, Tumoral and normal brain tissue extraction protocol for wide-scope screening of organic pollutants.

## Abstract

Little is known about the presence of organic pollutants in human brain (and even less in brain tumors). In this regard, it is necessary to develop new analytical protocols capable of identifying a wide range of exogenous chemicals in this type of samples (by combining target, suspect and non-target strategies). These methodologies should be robust and simple. This is particularly challenging for solid samples, as reliable extraction and clean-up techniques should be combined to obtain an optimal result. Hence, the present study focuses on the development of an analytical methodology that allows the screening of a wide range of organic chemicals in brain and brain tumor samples. This protocol was based on a solid-liquid extraction based on bead beating, solid-phase extraction clean-up with multi-layer mixed-mode cartridges, reconstitution and LC—HRMS analysis. To evaluate the performance of the extraction methodology, a set of 66 chemicals (e.g., pharmaceuticals, biocides, or plasticizers, among others) with a wide range of physicochemical properties was employed. Quality control parameters (i.e., linear range, sensitivity, matrix effect (ME%), and recoveries (R%)) were calculated and satisfactory results were obtained for them (e.g., R% within 60–120% for 32 chemicals, or ME% higher than 50% (signal suppression) for 79% of the chemicals).

## Graphical abstract

**Figure:**
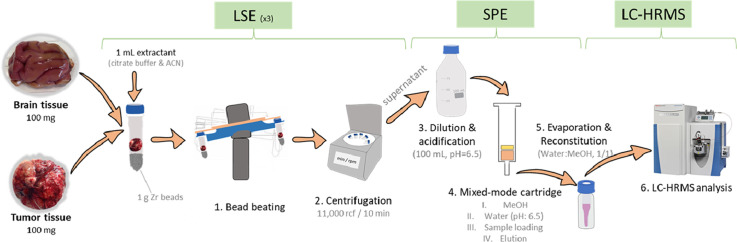

Specifications table

**Table:** 

Subject area:	Chemistry
More specific subject area:	Analytical chemistry, Molecular Biology and Environmental sciences Organic chemicals. Human biomonitoring.
Name of your protocol:	Tumoral and normal brain tissue extraction protocol for wide-scope screening of organic pollutants.
Reagents/tools:	Reagents and standards • Acetonitrile (HPLC-grade, Fischer Scientific) • Citric acid anhydrous (Scharlab) • Tri-Sodium citrate 2-hydrate (Sharlab) • Water (HPLC-grade, Fischer Scientific) • Distilled water (purification system, Aurium, PRO-VFT, Sartorius, Germany) • Ethyl acetate 99.6% (Acros Organics) • Methanol (HPLC-grade, Merck) • Ammonia solution 32% (Merck) • Formic acid 88–90% (Merck) • Ammonium acetate (Merck) • Standards and internal standards from Sigma-Aldrich (Steinheim, Germany) and LGC Standards (Barcelona, Spain). The IS used were clothianidin-d3, benzotriazole-d4, methyl paraben-d3, caffeine-d3, carbamazepine-d10, citalopram-d4, dimethyl phthalate-d5, metronidazole-d4, nicotine-d3, thiamethoxam-d3, venlafaxine-d6, and triphenyl phosphate-d15 Materials • Precision balance (Mettler Toledo) • Tissuelyzer FastPrep-24 5 G (MP Biomedicals) • Centrifuge 5424 (Eppendorf) • 2 mL extraction tubes (Deltalab) • Zirconium beads (Precellys) • Glass Pasteur pipettes • Glass tubes (> 3 mL) • Glass bottles (> 100 mL) • pH-meter • Mixed mode cartridges • Empty SPE tubes 6cc, Polypropylene (Phenomenex) • Frits for 6cc SPE tubes, 20 μm (Phenomenex) • Sepra ZT (30 μm, 85 A) ° powder (Phenomenex) • Sepra ZTL-WCX (100 μm, 300 A) ° powder (Phenomenex) • Sepra ZTL-WAX (115 μm, 330 A) ° powder (Phenomenex) • Isolute ENV+ powder (Biotage) • SPE glass column processor, J.T.Baker (VWR) • Vacuum pump • Glass tubes (> 6 mL) • ReactiVap (Thermo Scientific) • Nitrogen >99.9% (Linde Gas) • HPLC vials (Waters) • HPLC vials caps (Waters) • Micropipette (2–20 µL and 100–1000 µL) • Vortex agitator • 150 µL glass inserts
Experimental design:	A solid-liquid extraction based on bead beating was performed on human tissue (around 100 mg ww) using citrate buffer and acetonitrile as extractant (in triplicate). The extractant is diluted with water (100 mL) and pH was adjusted to 6.5 (using ammonia and formic acid) and passed through homemade mixed-mode cartridges previously conditioned with MeOH and H_2_O. Once eluted with organic solvent, the extract was dried under a stream of N_2_ (g) until dryness and reconstituted in methanol:water (1:1, *v/v*) for LC—HRMS analysis.
Trial registration:	Not applicable
Ethics:	The protocol of the biological surveillance program, number 07/2017, was reviewed and approved by the Ethical Committee for Clinical Research (CEIm) of the Pere Virgili Health Research Institute (IISPV), Reus/Tarragona, Spain, in March 20, 2017. Furthermore, the specific protocol for the biomonitoring study of autopsy tissues, number PR164/19, was complementarily evaluated and approved by the Clinical Research Ethics Committee (CEIC) of the Bellvitge University Hospital, Barcelona, Spain, on May 9, 2019. Adequate measures to ensure personal data protection and confidentiality were taken, according to the Regulation (EU) 2016/679 on the protection of natural persons with regard to the processing of personal data and on the free movement of such data, and the Spanish Law of Personal Data Protection and Digital Rights Guarantee (3/2018, of 5th December). We only used retrospective samples from deceased patients.
Value of the Protocol:	• Small amount of tissue required (from 50 mg ww) • Account for a wide spectrum of chemicals with different physicochemical properties, essential for nontarget analysis. • Simple protocol

## Description of protocol

### Background

The evaluation of the chemical exposome, defined as the total environmental exposure since conception hereinafter [1,2], is essential for assessing the potential health risk facing humanity [3]. Human biomonitoring (HBM) studies, making use of the last advances in high-resolution mass spectrometry (HRMS), have been usually applied to human biofluids such as urine, to some extent easy to sample [4], [5], [6], also requiring lower sample treatment. However, there is a current need for analytical methodologies that are capable to evaluate the chemical exposome in some other overlooked tissues, such as brain and tumor biopsies. The focused analysis of these matrices will improve our understanding on how organic pollutants enter and interact with brain tissues.

### Objective

The goal of the present study was to validate an analytical methodology to perform wide-scope target, suspect or non-target screenings of organic chemicals in brain samples from autopsies and tumor biopsies by liquid chromatography coupled to high-resolution mass spectrometry (LC—HRMS). A set of 66 chemicals were selected and used to evaluate the methodology. The selection was done based on diverse physicochemical properties (LogP between −0.2 and 6.3), presence of diverse heteroatoms (including S, P, Cl, Br, F…) and comprising different chemical classes (e.g., pharmaceuticals, perfluoroalkyl and polyfluoroalkyl substances (PFAS), biocides, UV-Filters, plastic additives, personal care products (PCPs), food related chemicals, anticorrosion agents, and flame retardants).

### Sampling campaign

Brain human tissue was obtained from HUB-IDIBELL Pathology Biobank and autopsies carried out at the Pere Virgili Health Research Institute (IISPV), Reus (Tarragona), during 2022. All samples were maintained at −80 °C until sample treatment and a pool of them (*n* = 5) was used for method validation.

### Sample treatment

The sample treatment procedure consisted of three different steps, based on solid-liquid extraction (SLE), solid-phase extraction clean-up (SPE) and reconstitution for LC—HRMS analysis. It is based in a previous methodology for the detection of emerging contaminants in biota described elsewhere [7].

I. Solid-liquid extraction (SLE)
  1. Fill extraction tubes with 1 g of zirconium beads.
  2. Add 100–150 mg wet weight (ww) (weight and note exact mass) of the sample into the extraction tube. Minimum recommended mass is 50 mg.
  3. Add surrogate standards solution (clothianidin-d3) to achieve a 50 µg L^−1^ in vial (in the final extract). The final volume extract (in µL) will be adjusted to the initial mass for each brain tissue (in mg ww). Allow evaporation of solvent (60 min, room temperature) so the standards will be better permeated into the matrix.
  4. Add 1 mL of the extractant. (See Note #1).
  5. SLE is now performed by bead beating, using FastPrep-24 5 G instrument (total time: 30 s, power: 5.5).
  6. Centrifuge the samples (11.000 g, 10 min).
  7. Transfer the supernatant into a labelled glass tube. These glass tubes require a volume capacity higher than 3 mL.
  8. Repeat steps 4–7 twice. All supernatant extracts from the same sample must be collected in the same glass tube.
  9. Eliminate the excess of organic solvent by evaporation using a N_2_ evaporator until half of the volume (1.5 mL aprox.). Select a flow that slightly breaks the surface tension of the sample.
  10. Transfer the extract to a glass bottle (>100 mL). To completely transfer sample extract, clean the glass tubes with 3 mL of water, vortex it and add it again to the glass bottle (three times).
  11. Add water up to 100 mL and adjust pH to 6.5 with ammonia and formic acid.
II. Solid phase extraction (SPE)
  1. Condition of homemade SPE cartridges (based on previous work [8]) (see Note #2) by gravity with a cartridge volume of methanol first and water (pH=6.5, ammonia and formic acid) then. Do not allow cartridge to completely dry.
  2. Load the sample (1 drop s^−1^ approx.) using a vacuum pump.
  3. Pass air through the cartridge (around 3 min) to dry it. If you cannot finish the protocol in one day, freeze the already dried cartridges until next day. Let them reach ambient temperature before following.
  4. Elute the cartridges in glass tubes in the following two steps:
    a. 4 mL of Mixture A (see Note #3). Then, pass air for 2 min.
    b. 2 mL of Mixture B (see Note #4). Then, pass air for 2 min.
III. Reconstitution
  1. Reduce the extracts with N_2_ up to less than 1 mL.
  2. Transfer the sample extracts from the glass tubes to HPLC vials. To achieve a quantitative transfer, add 200 μL of methanol to the glass tubes, vortex and transfer it again to the HPLC vials (three times).
  3. Bring HPLC vials until dryness with N_2_.
  4. Reconstitution in methanol: water (1:1, v/v) HPLC-grade. To avoid sample degradation, the reconstitution is performed in two steps:
    a. Firstly, the HPLC-grade methanol. In this point, vial can be frozen (recommendable at −80 °C). Follow a ratio 1:2 of volume of methanol (µL):weight of the sample (mg ww).
    b. The water is added the day samples are injected in the LC-HRMS. Follow a ratio 1:2 of volume of water (µL):weight of the sample (mg ww).

To control the instrument performance, isotopically labelled internal standards (IS) may be added also the injection day. These IS should be different from those previously added as surrogate.

**Note #1:** Preparation of extraction mixture.

1. Dissolve 2.8822 g of citric acid in 150 mL of water (S1).
2. Dissolve 1.4705 g of tri-sodium citrate 2-hydrate in 100 mL of HPLC water (S2).

Mix 118 mL of S1 with 82 mL of S2 and mix carefully. Then, add 200 mL volume of S1:S2 (59:41) mixture to 200 mL acetonitrile. Store the excess of the solutions in the fridge.

**Note #2:** Homemade cartridges contained: 0.2 g of Sepra ZT, 0.1 g Sepra ZTL-WCX, 0.1 g Sepra ZTL-WAX and 0.15 g of Isolute ENV+ from Biotage. The empty cartridge is filled with a frit, then 0.2 g of Sepra ZT, another frit, 0.35 g of a mix of the rest of the sorbents and the last frit. For more details see Gago-Ferrero et al. [8]

**Note #3:** Mixture A: Methanol (47% of the total volume), ethyl acetate (47% of the total volume),) and 32% ammonia solution (6% of the total volume).

**Note #4:** Mixture B: Methanol (49% of the total volume), ethyl acetate (49% of the total volume) and formic acid 88–90% (2% of the total volume).

### LC—HRMS analysis

Instrumental analysis was performed in an Acquity UHPLC system (Waters, Milford, USA) coupled to a Q-Exactive Orbitrap mass analyser (Thermo Fisher Scientific, Dreieich, Germany) by means of an electrospray ionization interface (ESI) in positive (ESI+) and negative (ESI-) modes. The chromatography and mass spectrometry parameters are described as follows:

I. Liquid chromatography
  • Column: Waters Cortecs C18 (2.1 × 100 mm, 2.7 μm)
  • Precolumn: Waters Cortecs C18 (2.1 × 5 mm, 2.7 μm)
  • Sample injected volume: 10 μl
  • Column temperature: 40 °C
  • Positive ionization mode (ESI+):
    - Mobile phase A: 0.1% formic acid in methanol
    - Mobile phase B: 0.1% formic acid in water
    - Gradient: (%A): Initial 5%, 75% at 7 min, 100% at 10 min, 100% at 15 min, 5% at 17 min and 5% at 23 min.
  • Negative ionization mode (ESI-):
    - Mobile phase A: 5 mM ammonium acetate in methanol
    - Mobile phase B: 5 mM ammonium acetate in water
    - Gradient: (%A): Initial 5%, 50% at 3 min, 90% at 6 min, 100% at 13 min, 100% at 17 min, 5% at 18 min, and 5% at 20 min.
II. Mass spectrometry
  • Spray voltage: 3000 V (ESI+) and 2800 V (ESI-)
  • Capillary temperature: 350 °C
  • Sheath gas: 40
  • Auxiliary gas flow: 10
  • Max. Spray current: 100
  • Probe heater temperature: 350 °C
  • S-Lens RF Level: 60

The instrument worked on data independent acquisition (DIA) mode. It consisted of a full scan with low collision energy and a full scan with a high collision energy (25 eV), in a mass-to-charge ratio (*m/z*) range from 67 to 1000 and resolving power of 60,000.

### Quality assurance and quality control

Quality assurance and quality control (QA/QC) measures were applied to prevent from any contamination during sample treatments or instrumental analysis. Thus, glass material was rinsed with distilled water and acetone and heated (450 °C) before use. Standards and internal standards were stored in amber glass vials at −20 °C in the dark to avoid degradation. Procedural blanks were done following the same steps of the protocol to account for any contamination. The 10 µg L^−1^ spiked point of the calibration curve of the pooled brain was injected every 10 injections to probe the repeatability of the signal. Methanol was injected every 10 injections to control possible carry-over issues. Internal standards were used as surrogate to control the sample treatment and some of them were added before injection to control the instrument performance and correct possible matrix effects.

### Method validation

A set of 66 chemicals were employed to evaluate the performance of the methodology, and the results are summarized in Table 1. These chemicals included pharmaceuticals, PFAS, biocides, UV-Filters, plastic additives, food related chemicals, anticorrosion agents, and flame retardants with different physicochemical properties (LogP in the range: −0.2 to 6.3). The proposed protocol has been validated in brain samples with a composite sample of brain by using the aforementioned 66 target chemicals . Due to the wide range of physicochemical properties of the selected compounds, this method is appropriate for non-target analysis.The following quality control parameters were assessed:

• Linearity. The linearity and linear range were evaluated with a calibration curve spiked at 0.1, 0.5, 1, 5, 10, 50, 100 µg L^−1^ in the pooled sample of brain (final extract). In addition, the same calibration curve (0.1, 0.5, 1, 10, 50, 100 µg L^−1^) was prepared in solvent (water/methanol, 1:1, *v/v*) to evaluate matrix effect.
• Matrix effect (ME%). The effect of the matrix was evaluated with the Eq. (1).
  (1) $$Matrix\;effect\;\left(\%\right)=100*\left(\frac{Slope\;in\;matrix}{Slope\;in\;solvent}\right)$$
  Values under/above 100% meant signal suppression/enhancement, respectively.
• Recovery (R%). A pooled sample was spiked at two concentration levels and analysed using the experimental protocol above. Fortification levels were 5 and 20 µg L^−1^ (theoretical concentration in vial, C_theoretical_). Additionally, 3 samples were processed without standard addition as matrix blanks. Then, recoveries were calculated with the Eq. (2) for each fortification level:
  (2) $$R\%=100*\frac{\left|Peak\;Area\;Spiked\right|-\left|Blanks\right|}{Slope\;*C_{theoretical}}\;$$
  where |Peak Area Spiked| referred to the average peak area of the spiked pooled sample at each fortification level, and |Blanks| referred to the average peak area of the matrix blanks. The slope was obtained from the calibration curve in matrix.
• Sensitivity. Method limits of quantification (LOQ) were estimated as the lowest observable peak in the calibration curve in matrix. And the limits of detection (LODs) were considered as 3/10 of the LOQs.

**Table 1:** Results of method validation including linear range, limit of quantification (LOQ), limit of detection (LOD), recovery (R%) and matrix effect (ME%), as well as ionization mode (IM).

Chemical	Class	LogP^a^	CAS	Linear range	LOQ (µg L^−1^)	LOD (µg L^−1^)	R^2^	R%^b^ (RSD%)	ME%	IM^d^
**Triethyl phosphate**	Flame retardant	0.8	78-40-0	0.5 - 100	0.5	0.15	0.989	130 (29)	20	+
**Tris(2-chloroethyl)phosphate**	Flame retardant	1.3	115-96-8	0.5 - 100	0.5	0.15	0.999	111 (3)	35	+
**4-Hydroxybenzoic acid**	Food related chemical	1.6	99-96-7	5 - 100	5	1.5	0.960	76 (49)	11	+
**4-Hydroxybenzoic acid-n-butyl ester**	Food related chemical	3.6	94-26-8	1 - 100	1	0.3	0.970	123 (7)	9	-
**Celestolide**	Food related chemical	5	13171-00-1	0.5 - 100	0.5	0.15	0.967	20 (127)	39	+
**Ethyl 3,4-Dihydroxybenzoate**	Food related chemical	1.8	3943-89-3	10 - 50	10	3	0.974	47 (57)	106	+ / -
**2,2′-Dihydroxy-4-methoxybenzophenone**	Industrial chemical	3.3	131-53-3	10 - 100	10	3	0.988	105 (9)	21	+ / -
**Benzotriazole**	Industrial Chemical	1	95-14-7	1 - 100	1	0.3	0.999	120 (9)	28	+
**Bisphenol G**	Industrial chemical	6.3	127-54-8	10 - 100	10	3	0.997	82 (8)	NA^c^	-
**Dimethylbenzotriazole**	Industrial Chemical	1.8	35899-34-4	0.5 - 100	0.5	0.15	0.999	76 (19)	28	+ / -
**Methylbenzotriazole**	Industrial Chemical	1.4	29878-31-7	1 - 100	1	0.3	0.995	122 (6)	26	+
**6:2 Fluorotelomersulfonate**	Industrial chemical (PFAS)	3.9	27619-97-2	10 - 100	10	3	0.969	137 (11)	10	-
**Perfluorobutanesulfonic acid**	Industrial chemical (PFAS)	2.3	375-73-5	0.1 - 100	0.1	0.03	0.966	134 (5)	5	-
**Benzyl paraben**	PCPs	3.6	94-18-8	0.5 - 100	0.5	0.15	0.984	130 (7)	7	-
**Isubutyl Paraben**	PCPs	3.4	2/3/4247	1 - 100	1	0.3	0.970	123 (7)	9	-
**Tonalide**	PCPs	5.3	21145-77-7	50 - 100	50	15	0.908	44 (114)	66	+
**Triclocarban**	PCPs	5.3	101-20-2	0.5 - 100	0.5	0.15	0.991	41 (10)	NA^c^	+ / -
**Umbelliferone**	PCPs	1.6	93-35-61	5 - 100	5	1.5	0.986	151 (42)	31	+
**Alachlor**	Pesticide	3.5	15972-60-8	10 - 100	10	3	0.962	48 (18)	14	+
**Atrazine-desethyl**	Pesticide	1.5	6190-65-4	1 - 100	1	0.3	0.993	105 (31)	22	+
**Dimethomorph**	Pesticide	3.9	110488-70-5	10 - 100	10	3	0.969	97 (22)	64	+
**Diuron**	Pesticide	2.7	330-54-1	5 - 50	5	1.5	0.998	44 (59)	23	+
**Flumequine**	Pesticide	2.9	42835-25-6	1 - 100	1	0.3	0.996	70 (13)	49	+
**Malathion**	Pesticide	2.4	121-75-5	10-100	10	3	0.983	2 (87)	44	+
**Metalaxyl**	Pesticide	1.6	57837-19-1	0.1 - 100	0.1	0.03	0.975	126 (18)	50	+
**Methiocarb**	Pesticide	2.9	2032-65-7	5 - 100	5	1.5	0.994	57 (21)	32	+
**Oxadiazon**	Pesticide	4.8	19666-30-9	5 - 100	5	1.5	0.992	22 (17)	51	+
**Oxathiapiprolin**	Pesticide	4.4	1003318-67-9	5-100	0.1	0.03	0.981	53 (37)	317	+
**Propanil**	Pesticide	3.1	709-98-8	5 - 100	5	1.5	0.973	48 (22)	20	+
**Sebuthylazine**	Pesticide	3.1	7286-69-3	5 - 100	5	1.5	0.996	57 (12)	NA^c^	+
**Tebuconazole**	Pesticide	3.7	107534-96-3	0.1 - 100	0.1	0.03	0.994	78 (3)	54	+
**Terbutylazine**	Pesticide	3.1	5915-41-3	5 - 100	5	1.5	0.996	68 (11)	38	+
**Zoxamide**	Pesticide	4.3	156052-68-5	10 - 100	10	3	0.951	56 (23)	43	+
**2-Hydroxy-5-octanoylbenzoic acid**	Pharmaceutical	5.2	78418-01-6	0.1 - 50	0.1	0.03	0.989	163 (31)	23	+ / -
**Atenolol**	Pharmaceutical	0.2	29122-68-7	1 - 100	1	0.3	0.99	85 (8)	44	+
**Carbamazepine**	Pharmaceutical	2.5	298-46-4	0.5 - 100	0.5	0.15	0.986	127 (8)	30	+
**Clarithromycin**	Pharmaceutical	3.2	81103-11-9	5-100	5	1.5	0.997	17 (37)	257	+
**Diclofenac**	Pharmaceutical	4.4	15307-86-5	0.1 - 100	0.1	0.03	0.989	103 (18)	47	+
**Enrofloxacin**	Pharmaceutical	-0.2	93106-60-6	1-100	1	0.3	0.988	24 (27)	226	+
**Ketoprofen**	Pharmaceutical	3.1	22071-15-4	5 - 100	5	1.5	0.971	124 (22)	38	+
**Lamotrigine**	Pharmaceutical	1.4	84057-84-1	50 - 100	50	15	0.942	18 (86)	19	+
**Mefenamic acid**	Pharmaceutical	5.1	61-68-7	0.1 - 100	0.1	0.03	0.993	67 (2)	49	+ / -
**Nalidixic acid**	Pharmaceutical	1.4	389-08-2	0.1 - 100	0.1	0.03	0.997	78 (4)	34	+
**Oxolinic acid**	Pharmaceutical	-0.2	14698-29-4	10 - 100	10	3	0.978	72 (29)	21	+
**Sulfadiazine**	Pharmaceutical	-0.1	68-35-9	0.5 - 100	0.5	0.15	0.992	106 (9)	35	+
**Sulfadimethoxine**	Pharmaceutical	1.6	122-11-2	0.5 - 100	0.5	0.15	0.995	76 (2)	27	+
**Sulfamerazine**	Pharmaceutical	0.1	127-79-7	1 - 100	1	0.3	0.997	72 (13)	33	+
**Sulfamethoxazole**	Pharmaceutical	0.9	723-46-6	1 - 100	1	0.3	0.995	72 (8)	23	+
**Sulfamethoxypyridazine**	Pharmaceutical	0.3	80-35-3	1 - 100	1	0.3	0.993	64 (9)	21	+
**Sulfaquinoxaline**	Pharmaceutical	1.7	59-40-5	0.1 - 100	0.1	0.03	0.994	74 (13)	30	+
**Sulfathiazole**	Pharmaceutical	0.1	72-14-0	5 - 100	5	1.5	0.991	71 (7)	23	+
**Tryptoline**	Pharmaceutical	1.5	16502-01-5	0.1 - 100	0.1	0.03	0.991	52 (71)	45	+
**Carbamazepine-10,11-epoxy**	Pharmaceutical TP^e^	1.3	36507-30-9	1 - 100	1	0.3	0.993	103 (6)	36	+
**N-acetyl sulfadiazine**	Pharmaceutical TP^e^	-0.2	127-74-2	10 - 100	10	3	0.995	58 (6)	41	+
**N-acetyl sulfamethazine**	Pharmaceutical TP^e^	0.1	100-90-3	1 - 100	1	0.3	0.997	88 (3)	44	+
**N-acetyl sulfapyridine**	Pharmaceutical TP^e^	-0.1	19077-98-6	1 - 100	1	0.3	0.991	93 (10)	29	+
**Bisphenol AF**	Plastic additive	4.5	1478-61-1	1 - 100	1	0.3	0.990	111 (11)	9	-
**Mono-cyclohexyl Phthalate**	Plastic additive	2.9	7517-36-4	0.5 - 100	0.5	0.15	0.970	117 (30)	95	+
**Mono(2-ethyl-5-hydroxyhexyl) Phthalate**	Plastic additive (metabolite)	2.5	40321-99-1	5 - 100	5	1.5	0.962	105 (29)	120	+
**Monobenzyl Phthalate**	Plastic additive (metabolite)	3.3	2528-16-7	5 - 100	5	1.5	0.980	114 (9)	53	+
**Benzophenone-1**	UV-filter	3.2	131-56-6	1 - 100	1	0.3	0.926	304 (8)	5	+ / -
**Benzophenone-2**	UV-filter	2.4	131-55-5	0.1 - 100	0.1	0.03	0.956	136 (28)	13	-
**Benzophenone-3**	UV-filter	3.6	131-57-7	0.1 - 100	0.1	0.03	0.993	123 (4)	28	+ / -
**Benzophenone-4**	UV-filter	2.2	4065-45-6	5 - 100	5	1.5	0.900	125 (8)	74	-
**4,4′-Dihydroxybenzophenone**	UV-filter metabolite	2.7	611-99-4	1 - 100	1	0.3	0.991	81 (22)	21	+ / -
**4-Hydroxybenzophenone**	UV-filter metabolite	3.1	1137-42-4	5 - 100	5	1.5	0.992	110 (21)	17	+

a LogP calculated by XLogP3 3.0 (PubChem release 2021.05.07).

b Recoveries were determined as the average at 5 and 20 (µg L^−1^).

c NA: data non-available as these chemicals were not spiked in the calibration curve in solvent.

d Ionization mode positive (+), negative (-) or both (+ / -).

e TP corresponds to Transformation Product.

### Method performance

The validation results are summarized in Table 1. Briefly, recoveries were satisfactory (in the range 60 – 120%) for 32 chemicals. Additional 13 chemicals showed recoveries between 120 and 150% and the rest provided low recovery values (17–60%). Malathion showed particularly low recovery (2%) and other 7 chemicals were not properly recovered as presented a RSD >40%. Regarding matrix effect, a high suppression was generally observed for all the chemicals, as expected for this complex matrix. Ninety-two percent of the tested chemicals showed signal suppression (79% showed suppression higher than 50%). However, the instrument response was good enough to achieve acceptable LOQs, with 59% of them ≤1 µg L^−1^, and almost 82% ≤5 µg L^−1^. All the chemicals presented a coefficient of determination (R^2^) higher than 0.90 in matrix, and 32 of them, higher than 0.99. Thus, the presented methodology provided satisfactory results for a wide number of chemicals with diverse physicochemical properties in a very complex human tissue (and small quantities of sample). This good method performance was remarkable considering the complexity of the evaluated matrix, and it was attributed to the sample pretreatment simplicity and comprehensiveness, which allowed to avoid compound losses. However, a reduced number of analytes were poorly recovered, showing high limits of quantification and a high matrix effect.

## CRediT authorship contribution statement

**Daniel Gutiérrez-Martín:** Formal analysis, Investigation, Data curation, Writing – original draft. **Montse Marquès:** Conceptualization, Methodology, Writing – review & editing, Supervision. **Albert Pons-Escoda:** Writing – review & editing, Resources, Funding acquisition, Supervision. **Noemi Vidal:** Conceptualization, Methodology, Writing – review & editing, Project administration, Funding acquisition. **Jordi Bruna:** Writing – review & editing, Resources, Funding acquisition. **Esteban Restrepo-Montes:** Writing – review & editing, Resources, Funding acquisition. **Rebeca López-Serna:** Writing – review & editing, Resources, Funding acquisition. **Francisco García-Sayago:** Writing – review & editing, Resources, Funding acquisition. **Carles Majos:** Writing – review & editing, Resources, Funding acquisition. **Pablo Gago-Ferrero:** Writing – review & editing, Resources, Funding acquisition. **Rubén Gil-Solsona:** Writing – review & editing, Resources, Funding acquisition.

## Declaration of Competing Interest

The authors declare that they have no known competing financial interests or personal relationships that could have appeared to influence the work reported in this paper.

## Data Availability

Data will be made available on request.
